# General self-efficacy and social support in men and women with pain – irregular sex patterns of cross-sectional and longitudinal associations in a general population sample

**DOI:** 10.1186/s12891-022-05992-5

**Published:** 2022-11-29

**Authors:** Anke Samulowitz, Gunnel Hensing, Inger Haukenes, Stefan Bergman, Anna Grimby-Ekman

**Affiliations:** 1 School of Public Health and Community Medicine, Institute of Medicine, The Sahlgrenska Academy, University of Gothenburg, Box 463, 40530 Gothenburg, Sweden; 2 Research Unit for General Practice, NORCE Norwegian Research Centre, Bergen, Norway; 3 Department of Global Public Health and Primary Care, University of Bergen, Bergen, Norway; 4 Spenshult Research and Development Centre, Halmstad, Sweden

**Keywords:** Sex, Psychosocial aspects, Social support, General self-efficacy, Gender roles

## Abstract

**Background:**

The study of sex and gender patterns in psychosocial resources is a growing field of interest in pain research with importance for pain rehabilitation and prevention. The aims of this study were first, to estimate cross-sectional differences in psychosocial resources (general self-efficacy and social support) across men and women in a population with frequent musculoskeletal pain (pain in the back or neck/shoulder nearly every day or now and again during the week for the last 12 months) and to compare these differences with a population with no frequent pain. Second, to examine if psychosocial resources at baseline were associated with pain at follow-up among men and women in the frequent pain population.

**Methods:**

This study was based on survey data from the Swedish Health Assets Project, including The General Self-Efficacy Scale and social support questions. Participants (*n* = 4010, 55% women) were divided into no frequent pain (*n* = 2855) and frequent pain (*n* = 1155). General self-efficacy and social support were analyzed (cross-sectional and longitudinal data) with linear and logistic regressions.

**Results:**

Men, with and without frequent pain, had higher general self-efficacy than the corresponding groups in women. Women, with and without frequent pain, had stronger emotional social support than the corresponding groups in men. Men with no frequent pain had weaker instrumental social support than women with no frequent pain (OR = 0.64 (95% CI 0.47–0.87)), men with frequent pain did not (OR = 1.32 (95% CI 0.86–2.01)). In the frequent pain population, the interaction between sex and strong (compared to weak) emotional social support was statistically significant (*p* = 0.040) for no frequent pain at follow-up, with women having OR = 1.81 and men OR = 0.62. Among women, strong emotional social support was associated with no frequent pain at follow-up. Among men, strong emotional social support was associated with frequent pain at follow-up.

**Conclusion:**

Some of the associations between general self-efficacy, social support and musculosceletal pain showed unexpected sex patterns. Gendered expectations might have relevance for some of the results.

**Supplementary Information:**

The online version contains supplementary material available at 10.1186/s12891-022-05992-5.

## Background

Most individuals with chronic pain need to find coping strategies, cognitive and behavioral ways to deal with their pain and a life altered by their pain [1, 2]. Coping strategies are based on psychosocial resources and affected by gendered expectations [3, 4], societal beliefs about how men and women are and how they are expected to behave [5]. Significantly more women than men suffer from chronic pain, and men and women cope with pain in different ways [1, 2, 6]. Women use a wider range of coping behaviors, tend to use more emotion-focused strategies and social support, and engage more than men in catastrophizing [4]. Men use more distractive and problem-focused behaviors and demonstrate higher self-efficacy [4]. Emotionality and social skills are associated with traditional femininity, whereas decisiveness and self-confidence are associated with traditional masculinity [3, 5]. In this study we examine general self-efficacy and social support. Differences between men and women, with and without pain, in self-efficacy and in social support have been shown [4, 7, 8] but little is known about the (prospective) associations between those psychosocial factors, sex/gender and musculoskeletal pain.

General self-efficacy (GSE) signifies an individual’s ability to believe in his or her capability to achieve goals and cope with stressful challenges [9]. GSE is closely connected to decisiveness, determination, control and self-confidence [10, 11], associated with traditional masculinity [12, 13], and men show higher GSE compared to women in general population studies [7, 14]. Pain research has shown a favorable effect of high self-efficacy on functional outcome [15], pain levels [16], pain-related disability [17], sick leave [18] and patients’ adherence to treatment recommendations [19]. However, it is not known yet, if men and women with and without pain show the same sex patterns in GSE or if men and women with pain benefit from high GSE in the same way.

In addition to GSE, social support is an important psychosocial resource, often defined as a backup available from others when needed [20]. It includes instrumental social support (ISS), assistance with daily life obligations, and emotional social support (ESS), to show concern, listen, care [20]. ESS is associated with emotionality and has been suggested as a way to express traditional femininity [3, 5]. ISS might be more difficult to categorize. It could either be associated with social support in the means of helping and caregiving, which usually is associated with femininity [3], or it could be associated with initiative and action, with often is associated with masculinity [3]. In pain research it is common not to separate ISS and ESS (e.g. [15, 21]) and it has been stated that women give and receive social support more than men [1, 2, 6]. However, some authors noticed (spousal) solicitousness, a social support-related construct [15, 22] that denotes giving attention, showing compassion, offering assistance or taking over obligations [22]. Studies indicate that social support might have a positive, stress-buffering effect, whereas solicitousness might have a negative effect on pain coping [15, 22]. Yet, there are knowledge gaps when it comes to potential sex differences in ISS and ESS among individuals with pain and little is known about sex differences in longitudinal associations between ISS, ESS and pain. In addition, the need to discuss if gendered expectations might have relevance for potential sex differences has been pointed out [2, 3, 6, 8].

The aims of this study were first to estimate cross-sectional sex differences in psychosocial resources (GSE, ISS, ESS) across men and women in a population with frequent pain. Second, to compare these differences with a population with no frequent pain and third, to examine associations between psychosocial resources at baseline and the likelihood of having no frequent pain at follow-up, among men and women with frequent pain at baseline.

## Methods

Data from the Health Assets Project (HAP) was used, a longitudinal cohort study with two data collections, carried out in February – April 2008 and September – November 2009 in Västra Götaland, Sweden. The project has been described in detail by Holmgren et al. [23]. Ethical approval was granted by the Regional Ethical Review Board of the University of Gothenburg in Sweden (registration number 039–08). Prior to the study, informed consent has been obtained from all participants. Our population-based study included a cross-sectional and a prospective-longitudinal part.

### Participants

The study was based on a random general population cohort (*n* = 7984). The response rate was 50.4%, while 49.6% did not respond to the invitation. A non-responder analysis of the HAP in an earlier study showed that non-participants were more likely to be men, born outside the Nordic countries, in the age-group 19–30 years, having low income, and living alone [24]. Seventeen participants did not answer questions about pain in the neck or back and the final study population consisted of 4010 participants, 2225 women (55%) and 1785 men. Characteristics of the study population are summarized in Table 1.

**Table 1:** Characteristics of the populations “frequent pain”, “no frequent pain” and “pain follow-up”

	Frequent pain		No frequent pain		Pain follow-up	
	Women n^1^ (%)	Men n (%)	Women n (%)	Men n (%)	Women n (%)	Men n (%)
**Total**	754 (65)	401 (35)	1471 (52)	1384 (48)	590 (67)	291 (33)
**Age**						
19–30 years	130 (17)	58 (15)	331 (23)	304 (22)	84 (14)	36 (12)
31–50 years	331 (44)	182 (45)	667 (45)	619 (45)	251 (43)	125 (43)
51–64 years	293 (39)	161 (40)	473 (32)	461 (33)	255 (43)	130 (45)
**Education**						
University/higher education	279 (37)	97 (24)	635 (43)	486 (35)	228 (39)	74 (25)
Upper secondary school	311 (41)	191 (48)	597 (41)	647 (47)	230 (39)	128 (44)
Compulsory school	154 (20)	111 (28)	227 (15)	235 (17)	123 (21)	87 (30)
[Missing]	10 (1)	2 (1)	12 (1)	16 (1)	9 (2)	2 (1)
**Country of birth**						
Nordic countries	666 (88)	350 (87)	1361 (93)	1250 (90)	526 (89)	259 (89)
Other countries	88 (12)	51 (13)	110 (8)	134 (10)	64 (11)	32 (11)
**Instrumental social support (ISS)**						
Strong ISS	402 (53)	218 (54)	928 (63)	858 (62)	319 (54)	162 (56)
Mixed ISS	252 (33)	133 (33)	442 (30)	364 (26)	203 (34)	97 (33)
Weak ISS	87 (12)	39 (10)	79 (5)	123 (9)	61 (10)	24 (8)
[Missing]	13 (2)	11 (3)	22 (2)	39 (3)	7 (1)	8 (3)
**Emotional social support (ESS)**						
Strong ESS	606 (80)	270 (67)	1308 (89)	1098 (79)	473 (80)	203 (70)
Mixed ESS	75 (10)	69 (17)	92 (6)	146 (11)	61 (10)	48 (17)
Weak ESS	59 (8)	52 (13)	55 (4)	109 (8)	46 (8)	33 (11)
[Missing]	14 (2)	10 (3)	16 (1)	31 (2)	10 (2)	7 (2)
**General self-efficacy (GSE), dichotomized**						
High general self-efficacy	445 (59)	276 (69)	1099 (75)	1125 (81)	354 (60)	202 (69)
Low general self-efficacy	296 (39)	115 (29)	346 (24)	239 (17)	227 (39)	84 (29)
[Missing]	13 (2)	10 (3)	26 (2)	20 (1)	9 (2)	5 (2)
	Women mean (SD^2^)	Men mean (SD)	Women mean (SD)	Men mean (SD)	Women mean (SD)	Men mean (SD)
**General self-efficacy (GSE), continuous**	2.8 (0.53)	2.9 (0.48)	3.0 (0.42)	3.1 (0.43)	2.8 (0.52)	2.9 (0.47)

“Frequent pain”: Pain in the back or neck/shoulder, nearly every day or now and then during the week during the past 12 months.” No frequent pain”: Pain in the back or neck/shoulder, now and then during the month or almost never or never during the past 12 months. “Pain follow-up”: Individuals with frequent pain at baseline who rated their pain in the back or neck/shoulder at follow-up.

^1^*n* number, ^2^*SD* standard deviation

**Fig. 1:**
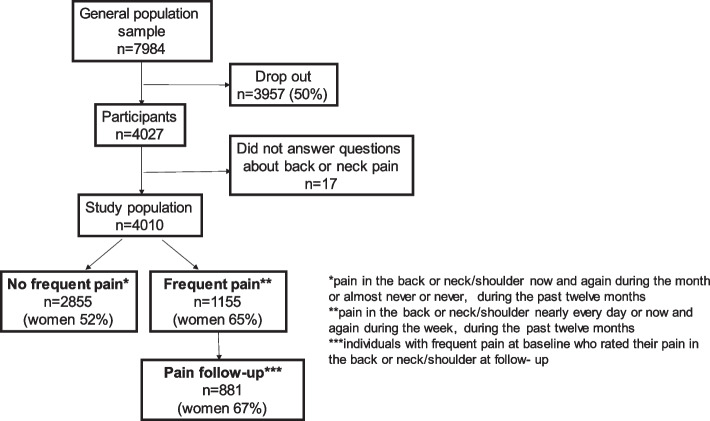
General population sample from the Swedish Health Assets Project (HAP), study population, including three subpopulations

In the survey the participants were asked “How often have you had the following symptoms during the past twelve months?”. Back pain and neck/shoulder pain were listed among twelve different symptoms. Possible answers were “nearly every day”, “now and again during the week”, “now and again during the month” or “almost never or never”. The study population, see fig. 1, was divided into two subpopulations:

1. **Frequent pain**. Individuals reporting back pain or neck/shoulder pain nearly every day or now and again during the week, during the past 12 months (*n* = 1155, 65% women).
2. **No frequent pain**. Individuals reporting back pain or neck/shoulder pain now and again during the month or almost never or never, during the past 12 months (*n* = 2855, 52% women).
3. **Pain follow-up**. Individuals with frequent pain at baseline who rated their pain in the back or neck/shoulder at follow-up (*n* = 881, 67% women).

### Instruments

*The General Self-Efficacy scale* (GSE scale) is a psychometric 10-item scale, rated at a four-point Likert scale [25]. The Swedish version of the GSE scale was validated in HAP in 2012 [14]. The mean score was used for statistical analyses.

**Fig. 2:**
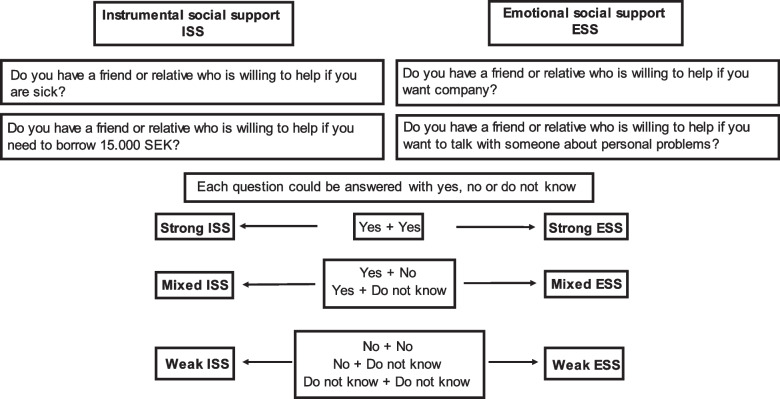
Instrumental and emotional social support. Questions used and categorization into strong, mixed and weak support

*Social support* was measured with four questions, see Fig. 2. The questions have earlier been used, for example in the ENRICHD-study (Enhancing Recovery in Coronary Heart Disease) [26]. We combined the scores from two of the four questions to the variable instrumental social support (ISS) and the scores from the other two questions were combined to the variable emotional social support (ESS). Within each variable, the scores were divided into three categories: strong, mixed and weak ISS, and strong, mixed and weak ESS, see Fig. 2.

### Statistical analysis

The IBM SPSS statistics, version 27 was used for descriptive statistics, linear, multinomial logistic and binary logistic regressions. Cross-sectional data was used to estimate sex differences in psychosocial resources for the subpopulations frequent pain and no frequent pain. Longitudinal data was used to examine associations between psychosocial resources at baseline (subpopulation frequent pain) and the likelihood of having no frequent pain at follow-up (subpopulation pain follow-up). The subpopulations are described in Fig. 1.

To estimate sex differences in psychosocial resources in the frequent pain population and to compare these differences with a no frequent pain population we used linear regression to examine GSE (with GSE as a continuous dependent variable) and a multinomial logistic regression to examine ISS and ESS (with three category dependent variables).

To examine the associations between baseline psychosocial resources and no frequent pain at follow-up, we used binary logistic regression. In these models, GSE was dichotomized into a 25/75% distribution with a cut-off at 2.70. Low GSE was represented by the lowest 25% (1.00–2.70), high GSE by 26–100% (2.71–4.00) The cut-off was based on an earlier study on GSE and mental health in the HAP [27].

Results are presented unadjusted and adjusted for age, level of education and country of birth. Significance level was set at *p* < 0.05. In the logistic regression models, predictors were tested using likelihood-ratio tests for the models’ partial effects. From these models also estimations of odds ratio (OR) and 95% Wald Confidence Interval (CI) were presented.

## Results

### The distribution of psychosocial resources among men and women with frequent and no frequent pain

#### Differences between men and women in GSE (general self-efficacy)

Men had higher GSE than women, in both the frequent pain group (mean 2.9 (SD 0.48) vs 2.8 (SD 0.53); *p* < 0.001) and the no frequent pain group (mean 3.1 (SD 0.43) vs 3.0 (SD 0.42); p < 0.001) (Table 2). The range of GSE was 1–4 and the size of the sex differences was similar in the frequent pain and the no frequent pain group.

**Table 2:** Associations between sex and general self-efficacy (GSE), frequent and no frequent pain, linear regression model

	Unadjusted models			Adjusted models (age)			Adjusted models (age, education)			Adjusted models (age, education, place of birth)		
	B^1^	95% CI^2^	*p*-value	B	95% CI	*p*-value	B	95% CI	*p*-value	B	95% CI	*p*-value
**Frequent pain**												
Intercept	2.8	2.76–2.83	< 0.001	2.9	2.81–3.03	< 0.001	2.7	2.64–2.93	< 0.001	2.6	2.39–2.71	< 0.001
Men (women = ref^3^)	0.10	0.04–0.17	**0.001**	0.11	0.04–0.17	**0.001**	0.12	0.06–0.18	**< 0.001**	0.12	0.06–0.18	**< 0.001**
**No frequent pain**												
Intercept	3.0	2.94–2.98	< 0.001	3.0	2.96–3.08	< 0.001	2.9	2.84–3.00	< 0.001	2.9	2.84–3.03	< 0.001
Men (women = ref)	0.10	0.07–0.14	**< 0.001**	0.10	0.07–0.14	**< 0.001**	0.11	0.08–0.14	**< 0.001**	0.11	0.08–0.14	**< 0.001**

^1^*B* parameter estimate, ^2^*CI* confidence interval, ^3^*ref* reference group

#### Differences between men and women in ISS (instrumental social support)

In the *no frequent pain group*, the odds for men considering strong ISS (OR = 0.64 (95% CI 0.47–0.87)) and mixed ISS (OR = 0.54 (95% CI 0.39–0.74) were statistically significant lower compared to women. For the *frequent pain group*, the odds for men considering strong ISS (OR = 1.32 (95% CI 0.86–2.01)) and mixed ISS (OR = 1.19 (CI 0.76–1.85) were not statistically significant higher compared to women. The interaction between sex and pain group was statistically significant for strong ISS (no frequent pain OR = 0.64, frequent pain OR = 1.33) and mixed ISS (no frequent pain OR = 0.54, frequent pain OR = 1.19), men compared to women (Table 3). A supplementary table where controlling variables have been added stepwise is also provided (Supplementary Table 1).

**Table 3:** Associations between sex, instrumental social support (ISS) and emotional social support (ESS), multinomial linear regression

	Unadjusted models			Adjusted models (age, education, place of birth)		
	OR^1^	95% CI^2^	*p*-value	OR	95% CI	*p*-value
**Instrumental social support**						
*Frequent pain*						
Strong ISS Men (women = ref^3^)	1.21	0.80–1.83	0.365	1.32	0.86–2.01	0.205
Mixed ISS Men (women = ref)	1.18	0.76–1.81	0.459	1.19	0.76–1.85	0.445
*No frequent pain*						
Strong ISS Men (women = ref)	0.59	0.44–0.80	**0.001**	0.64	0.47–0.87	**0.004**
Mixed ISS Men (women = ref)	0.53	0.39–0.73	**< 0.001**	0.54	0.39–0.74	**< 0.001**
*Total population*						
Strong ISS Men (women = ref)	1.21	0.80–1.83	0.365	1.33	0.87–2.03	0.187
No frequent pain (frequent pain = ref)	2.54	1.83–3.52	**< 0.001**	2.24	1.60–3.13	**< 0.001**
Sex (male)*^4^ Pain (no frequent)	0.49	0.30–0.82	**0.006**	0.48	0.29–0.81	**0.006**
Mixed ISS Men (women = ref)	1.18	0.76–1.81	0.459	1.19	0.77–1.84	0.439
No frequent pain (frequent pain = ref)	1.93	1.83–3.52	**< 0.001**	1.82	1.28–2.57	**0.001**
Sex (male)* Pain (no frequent)	0.45	0.30–0.82	**0.003**	0.46	0.27–0.78	**0.005**
**Emotional social support** *Frequent pain*						
Strong ESS Men (women = ref)	0.51	0.34–0.75	**0.001**	0.43	0.31–0.61	**< 0.001**
Mixed ESS Men (women = ref)	1.04	0.64–1.71	0.865	0.81	0.53–1.24	0.329
*No frequent pain*						
Strong ESS Men (women = ref)	0.42	0.30–0.59	**< 0.001**	0.50	0.33–0.75	**0.001**
Mixed ESS Men (women = ref)	0.80	0.53–1.21	0.295	1.00	0.60–1.65	0.989
*Total population*						
Strong ESS Men (women = ref)	0.51	0.34–0.75	**0.001**	0.51	0.34–0.77	**0.001**
No frequent pain (frequent pain = ref)	2.32	1.58–3.39	**< 0.001**	2.09	1.42–3.08	**< 0.001**
Sex (male)* Pain (no frequent)	0.84	0.50–1.41	0.506	0.85	0.50–1.45	0.560
Mixed ESS Men (women = ref)	1.04	0.64–1.71	0.865	1.00	0.61–1.65	0.998
No frequent pain (frequent pain = ref)	1.32	0.82–2.12	0.260	1.25	0.77–2.03	0.370
Sex (male)* Pain (no frequent)	0.77	0.40–1.47	0.422	0.82	0.43–1.57	0.546

^1^*OR* odds ratio, ^2^*CI* confidence interval, ^3^*ref* reference group, ^4^* interaction

Within the *no frequent pain group,* the prevalence ratio for weak ISS was 66% *higher* for men compared to women. Within the *frequent pain group,* this ratio was 14% *lower* for men compared to women (Table 4).

**Table 4:** Prevalence of weak ISS among men and women, stratified into no frequent / frequent pain

	Weak instrumental social support	Prevalence (%)	Prevalence ratio Men/Women
No frequent pain	Men	9.1	1.66
	Women	5.5	
Frequent pain	Men	10.0	0.86
	Women	11.7	

Analyses are based on the logistic regression presented in Table 3

#### Differences between men and women in ESS (emotional social support)

In the *no frequent pain* and the *frequent pain group* the odds for men considering strong ESS were statistically significant lower compared to women (no frequent pain OR = 0.50 (95% CI 0.33–0.75); frequent pain OR = 0.43 (95% CI 0.31–0.61)) (Table 3).

### Psychosocial resources at baseline and pain at follow-up

Sex alone (analyzed without social resources) was not a statistically significant predictor for frequent pain at follow-up. Moreover**,** no statistically significant associations between GSE at baseline and no frequent pain at follow-up were found, neither for men nor women (Table 5).

**Table 5:** Prospective associations between psychosocial factors at baseline and no frequent pain at follow-up

	Unadjusted models			Adjusted models (age, education, place of birth)		
	OR^1^	95% CI^2^	*p*-value	OR	95% CI	*p*-value
**GSE (general self-efficacy)**						
Men (women = ref^3^)	0.94	0.70–1.26	0.677	1.01	0.75–1.37	0.931
High GSE (low = ref)	0.75	0.56–1.01	0.059	0.80	0.59–1.08	0.149
*Men*						
High GSE (low = ref)	0.74	0.43–1.27	0.284	0.78	0.44–1.36	0.378
*Women*						
High GSE (low = ref)	0.76	0.53–1.07	0.119	0.81	0.57–1.17	0.261
**ISS (instrumental social support)**						
Men (women = ref)	0.97	0.72–1.31	0.856	1.03	0.76–1.40	0.839
ISS strong (weak = ref)	2.03	1.21–3.55	**0.009**	1.71	1.01–3.02	0.054
ISS mixed (weak = ref)	1.86	1.08–3.30	**0.029**	1.73	1.00–3.10	0.057
*Men*						
ISS strong (weak = ref)	1.72	0.68–4.96	0.278	1.35	0.51–4.01	0.562
ISS mixed (weak = ref)	1.85	0.70–5.48	0.233	1.62	0.60–4.88	0.360
*Women*						
ISS strong (weak = ref)	2.19	1.19–4.28	**0.016**	1.89	1.01–3.74	0.056
ISS mixed (weak = ref)	1.85	0.97–3.69	0.070	1.74	0.91–3.52	0.106
*With interaction*						
Men (women = ref)	1.12	0.35–3.27	0.841	1.23	0.38–3.65	0.710
ISS strong (weak = ref)	2.19	1.19–4.28	**0.016**	1.87	1.00–3.69	0.059
ISS mixed (weak = ref)	1.85	0.97–3.69	0.070	1.74	0.91–3.50	0.107
ISS (strong)*^4^Sex (male)	0.78	0.25–2.65	0.683	0.75	0.24–2.56	0.634
ISS (mixed)*Sex (male)	1.00	0.31–3.52	0.997	0.96	0.29–3.41	0.951
**ESS (emotional social support)**						
Men (women = ref)	0.98	0.73–1.32	0.902	1.03	0.76–1.40	0.069
ESS strong (weak = ref)	1.23	0.76–2.06	0.412	1.14	0.69–1.92	0.623
ESS mixed (weak = ref)	1.36	0.74–2.52	0.330	1.34	0.72–2.50	0.360
*Men*						
ESS strong (weak = ref)	0.67	0.32–1.44	0.293	0.60	0.28–1.30	0.187
ESS mixed (weak = ref)	1.06	0.43–2.61	0.906	1.07	0.43–2.70	0.881
*Women*						
ESS strong (weak = ref)	1.94	0.99–4.09	0.065	1.82	0.92–3.88	0.101
ESS mixed (weak = ref)	1.67	0.72–4.05	0.242	1.63	0.69–4.00	0.275
*With interaction*						
Men (women = ref)	2.34	0.90–6.29	0.084	2.51	0.95–6.86	0.067
ESS strong (weak = ref)	1.94	0.99–4.09	0.065	1.81	0.91–3.87	0.102
ESS mixed (weak = ref)	1.67	0.72–4.05	0.242	1.63	0.69–4.00	0.276
ESS (strong)*Sex (male)	0.35	0.12–0.96	**0.042**	0.34	0.12–0.94	**0.040**
ESS (mixed)*Sex (male)	0.63	0.18–2.18	0.468	0.66	0.19–2.33	0.520

Binary logistic regression was used to model the prevalence of no frequent pain at follow-up among men and women with frequent pain at baseline. Predictors in the model: General self-efficacy (GSE), instrumental social support (ISS) and emotional social support (ESS) at baseline, respectively, and sex. Interaction included

^1^*OR* odds ratio, ^2^*CI* confidence interval, ^3^*ref* reference group, ^4^* interaction

Individuals reporting strong ISS or mixed ISS at baseline had statistically significantly higher odds of having no frequent pain at follow-up (strong ISS crude OR = 2.03 (95% CI 1.21–3.55)); mixed ISS crude OR = 1.86 (95% CI 1.08–3.30)). However, after adjustment for covariates the ORs were decreased and not statistically significant, though with CIs still indicating an association (strong ISS OR = 1.71 (95% CI 1.01–3.02); mixed ISS OR = 1.73 (95% CI 1.00–3.10)) (Table 5).

Separate analyses for men and women showed that the crude association between strong ISS at baseline and no frequent pain at follow-up was statistically significant only for women (strong ISS OR = 2.19 (95% CI 1.19–4.28)). In the adjusted analysis there was no statistical significance in the likelihood-ratio tests for the model’s partial effects, but a small decrease in the OR (1.89) and a CI still indicating an association.

For no frequent pain at follow-up the interaction between sex and strong ESS (reference: weak) was statistically significant, with women having OR = 1.81 and men OR = 0.62 (Table 5). Prevalence ratio showed that women with strong ESS at baseline (compared to weak ESS) had a 55% *higher* chance of no frequent pain at follow-up. Men with strong ESS at baseline (compared to weak ESS) hade 28% *lower* chance of having no frequent pain at follow-up. Men with weak ESS at baseline had 39% higher chance of having no frequent pain at follow-up (Table 6).

**Table 6:** Prevalence no frequent pain at follow-up, among men and women with frequent pain at baseline

	Strong ESS Prevalence, 95% CI^1^ Adjusted [Unadjusted]	Mixed ESS Prevalence, 95% CI Adjusted [Unadjusted]	Weak ESS Prevalence, 95% CI Adjusted [Unadjusted]	Prevalence ratio of strong/weak ESS Adjusted [Unadjusted]
Men	28 (21–36) [33 (27–40)]	41 (27–56) [44 (31–58)]	39 (24–57) [42 (27–60)]	0.72 [0.79]
Women	31 (26–38) [38 (34–42)]	29 (19–42) [34 (24–47)]	20 (11–34) [24 (14–38)]	1.55 [1.58]

Analyses are based on the binary logistic regression presented in Table 5. Results are stratified in groups of strong, mixed or weak emotional social support (ESS) at baseline. Prevalence and prevalence ratio are presented

^1^*CI* confidence interval

## Discussion

The main findings were that while sex differences in GSE and ESS were similar in the frequent pain and the no frequent pain group, sex patterns for ISS differed between the frequent pain group and the no frequent pain group. Women with no frequent pain had stronger ISS than men, women with frequent pain did not. In addition, for women with frequent pain, strong ISS at baseline was associated with no frequent pain at follow-up. Even strong ESS at baseline was associated with no frequent pain at follow-up for women with frequent pain. Contrary to women, for men, *weak* ESS at baseline was associated with no frequent pain at follow-up. Even though the effect sizes varied throughout the study, different sex patterns emerged in the associations between psychosocial resources and frequent pain.

### Similar sex patterns in GSE for individuals with frequent and no frequent pain

This study showed that men with no frequent pain had higher GSE than women. The effect sizes were small but in line with earlier research [7]. Previous studies have also shown higher pain-specific self-efficacy in men, compared to women [28], but, to our knowledge, this is the first study that showed similar sex patterns in GSE for individuals with frequent and no frequent pain. It has been proposed that self-efficacious individuals engage more actively in coping strategies [11] and adjust better to chronic pain [29], leading to lower perceived pain intensity [10, 29], better physical functioning [10, 30], lower pain-disability [17] and an overall positive effect on pain [10, 15]. In addition, in a Swedish study from 2018, women with chronic pain and high GSE reported higher levels of well-being than women with low GSE [31]. Men and women with frequent pain seem to benefit from higher GSE [10, 15, 18, 28, 30, 31] and it should be further discussed how to address GSE in pain rehabilitation, not least for women [18, 28, 31].

Higher GSE in men and lower GSE in women in the general population has been explained by gendered expectations [32]. In our study, men and women with frequent pain had statistically significant lower GSE than men and women with no frequent pain. Lower GSE in men with frequent pain can be discussed in relation to a perceived loss of masculinity. Bernardes & Lima (2010) found that men with chronic pain were perceived by nurses and laypeople as less masculine compared to men without pain [33]. Men with chronic pain have also reported perceived expectations from society and health care personnel that “real men” should not feel or show pain, thereby questioning their masculinity [5, 34]. It is possible that men with frequent pain experience that they cannot live up to the expected role of a masculine man [5], which could be one possible explanation of their lower GSE.

A different reasoning might explain why women with frequent pain had lower GSE than women with no frequent pain. It has been argued that individuals with low GSE tend to develop an even lower GSE when faced with failures [35]. A review of gender norms in pain literature identified gender patterns, illustrating that women with chronic pain not only may regard their chronic pain as a failure but also may perceive it as a personal failure when they are not taken seriously by family members or health care personnel [5]. If women with low GSE initially also perceived their pain as a weakness, this might have led to even lower reports of GSE in women with frequent pain.

### Women with frequent pain reported weak ISS

In this study, women with frequent pain did not have stronger ISS than men with frequent pain. This was a surprising result, as earlier studies have reported that women with pain generally give and receive more social support than men [1, 2, 6]. Our study showed that it might be appropriate to differentiate between men’s and women’s ESS and ISS, and not to assume that women with pain receive social support of different kinds to a greater extent than men.

One of the main results were that women with frequent pain had stronger ESS than men with frequent pain but did not have stronger ISS. Gendered expectations might have played a role for differences in ESS and ISS for women with frequent pain. ESS is associated with emotionality and has been suggested as a feminine trait [3], which might be the reason why women with no frequent pain, as well as women with frequent pain reported higher ESS than men in our study. ISS could either be associated with social support in the means of helping and caregiving, which is associated with femininity [3], or it could be associated with initiative and action, with is associated with masculinity [3]. The ambiguity of ISS as an expression for typical femininity may become visible first in times when more ISS is needed, for example when a person suffers from pain. This could explain why women with no frequent pain, with less need of ISS, perceived their access to ISS as higher than women with frequent pain.

### For women with frequent pain, strong ISS and ESS were associated with no frequent pain at follow-up

The positive effect of strong social support for women’s sickness absence [36] and depression [37] has been discussed earlier. Eight out of 13 studies in a systematic review showed that weak social support was a predictor of depression for women only [37]. For women who are overrepresented in both chronic pain and depression, strong social support seems to be associated with less depression and less frequent pain.

Our results showed associations between strong ISS, strong ESS and no frequent pain prospectively, especially for women. Some researchers mean that social support can have a stress-buffering effect and that this effect is visible first in stressful times [8, 20–22]. Especially women, with greater family and household obligations may experience additional stress to an already stressful situation when they are in pain, and they may gain more than men from the stress-buffering effect of social support. Grav et al.’s (2012) research on social support and depression led to the conclusion that ESS is more important for women and ISS is more important for men [38]. In our results both ISS and ESS predicted no frequent pain for women, but not for men, implying that, contrary to Grav et al., strong ESS and ISS may be especially important for women. Increased consciousness about gendered living conditions, as well as gendered expectations about social support, in pain research and clinical practice, might hold the potential to improve pain research, treatment and prevention.

### For men with frequent pain, strong ESS was associated with frequent pain at follow-up

Contrary to women, men with frequent pain and strong ESS at baseline were more likely to have frequent pain at follow-up. It has been proposed that another aspect of ESS, (spousal) solicitousness, might lead to increased pain [22, 39]. For instance, Fillingim et al. (2003) showed that spousal solicitousness was associated with increased pain in men and women but more consistent among men [40]. In addition, solicitousness was also associated with higher self-reported disability in men but not in women [40]. Even in our results, there was an association between strong ESS at baseline and frequent pain at follow-up for men but not for women. It is possible that men who rated high on ESS also perceived solicitousness. Another possibility is that strong ESS may have encouraged men with frequent pain to allow themselves to feel the pain and admit their needs, an effect earlier described related to sickness absence [36]. However, it should be further explored how men perceive ESS in different circumstances and how they can benefit from ESS in the best possible way. It should also be further explored how ESS and (spousal) solicitousness are related and if this relation, consistently over study populations, is different for men and women and affected by gendered expectations.

### Methodological considerations

This study has some limitations that should be addressed. The response rate was 50.4%, which implicates a risk for selection bias. A non-responder analysis of the study population showed that non-participants were more likely to be men, born outside the Nordic countries, in the age-group 19–30 years, having low income, and living alone [24]. It is possible that the drop-out among men included more men with frequent pain than no frequent pain, which might have led to an overestimation of sex differences in the prevalence of frequent pain. But even a possible overestimation of sex differences in pain prevalence would probably not have changed our results on sex differences in the associations between psychosocial resources and pain.

Measuring social support is a challenge and different scales are used in studies [8], making the comparison of our study with other studies difficult. Nonetheless, the four questions used in our study are chosen from the ENRICHD Social Support Inventory (ESSI), a 7-item measure, developed as a screening tool, whose validity and reliability has been demonstrated [41].

GSE was not a predictor for frequent or no frequent pain, neither for men nor women. Our results might have been different if we had used pain-specific self-efficacy instead of GSE. But even if the greater preciseness of domain-specific self-efficacy measurements has been discussed, GSE scales have also proved to be a valid and useful measurement of self-efficacy [11], and our results on sex differences in GSE within the frequent pain group are in line with sex differences in pain self-efficacy shown by Ferrari et al. [28].

The present study is based on self-reported psychosocial resources and there is a possibility of reporting bias. It has been argued that, as part of gendered expectations, women are expected to provide to and receive social support from other women, whereas men mainly receive social support from women [20]. But even if men receive social support, part of conventional masculine gender expectations is to show independence and deny the need of help [3, 42]. Our results might have been different if we had measured received instead of perceived social support. However, the effect of received social support has been more inconsistent across studies, compared to the consistently reported beneficial effect of perceived social support [8].

Finally, we controlled for the covariates age, level of education and country of birth but other factors like mental well-being, marital status or other personal relationships may have influenced our results. In addition, we assessed the distribution of psychosocial resources among men and women but do not know if there were any participants identifying themselves as non-binary.

The study has several strengths worth mentioning. The longitudinal design made it possible to estimate differences between men and women with frequent and no frequent pain at baseline, and to analyze associations between psychosocial factors, sex and pain prospectively. In addition, this study is based on a large, random population-based sample, making the results generalizable to the general population and to provide new, complementary knowledge to clinical pain research.

## Conclusion

Although it has been discussed earlier that women receive more social support than men [8], this is, to the best of our knowledge, the first study that found differences in the prevalence of ISS and ESS among women with frequent pain. The results also showed different sex patterns for ISS and ESS as predictors for frequent pain. Men and women with pain might benefit from differentiated assessments of instrumental and emotional social support, as well as increased consciousness about gendered expectations attached to social support. Potential sex and gender differences in ISS and ESS should be further explored in public health studies and clinical pain research. Further research should also expand the knowledge about gendered expectations to transgender, intersex and non-binary individuals. Psychosocial resources are important for men’s and women’s use of coping strategies. Still, in current pain research there is a focus on biological and individual psychological factors and there is a knowledge gap about the importance of social factors [43]. Our results showed the need to further explore the complex relations between sex, gender, social support and musculoskeletal pain.

## Supplementary Information

**Additional file 1.**

## Data Availability

The data that support the findings of this study are available from “Swedish National Data Service” (SND 0870), but restrictions apply to the availability of these data, which were used under license for the current study, and so are not publicly available. Data are however available from the authors upon reasonable request and with permission of “Swedish National Data Service”.

## Abbreviations

GSE: General self-efficacy
ISS: Instrumental social support
ESS: Emotional social support
HAP: Health Assets Project
