# Supplementary material for: General self-efficacy and social support in men and women with pain – irregular sex patterns of cross-sectional and longitudinal associations in a general population sample
Source: BMC Musculoskelet Disord. 2022 Nov 29;23:1026. doi: 10.1186/s12891-022-05992-5 (PMC9707264; doi:10.1186/s12891-022-05992-5)
Supplement: Supplementary file 1 — Additional file 1. [file 12891_2022_5992_MOESM1_ESM.docx]

# Additional file 1

**Supplementary table 1.** Associations between sex, instrumental and emotional social support, multinomial linear regression, controlling variables included

|  |  |  |  |  |  |  |  |  |  |  |  |  |  |  |  |  |
| --- | --- | --- | --- | --- | --- | --- | --- | --- | --- | --- | --- | --- | --- | --- | --- | --- |
|  |  | Unadjusted model | | |  | Adjusted model 1  (age) | | |  | Adjusted model 2  (age, education) | | |  | Adjusted model 3  (age, education, place of birth) | | |
|  |  | OR^1^ | 95% CI^2^ | p-value |  | OR | 95% CI | p-value |  | OR | 95% CI | p-value |  | OR | 95% CI | p-value |
| **Instrumental social support**  *Frequent pain* |  |  |  |  |  |  |  |  |  |  |  |  |  |  |  |  |
| Strong ISS Men (Women=ref^3^) |  | 1.21 | 0.80-1.83 | 0.365 |  | 1.22 | 0.81-1.85 | 0.339 |  | 1.32 | 0.87-2.00 | 0.200 |  | 1.32 | 0.86-2.01 | 0.205 |
| Mixed ISS Men (Women=ref) |  | 1.18 | 0.76-1.81 | 0.459 |  | 1.19 | 0.77-1.84 | 0.429 |  | 1.19 | 0.77-1.84 | 0.437 |  | 1.19 | 0.76-1.85 | 0.445 |
| *No frequent pain* |  |  |  |  |  |  |  |  |  |  |  |  |  |  |  |  |
| Strong ISS Men (Women=ref) |  | 0.59 | 0.44-0.80 | **0.001** |  | 0.60 | 0.45-0.81 | **0.001** |  | 0.62 | 0.46-0.84 | **0.002** |  | 0.64 | 0.47-0.87 | **0.004** |
| Mixed ISS Men (Women=ref) |  | 0.53 | 0.39-0.73 | **<0.001** |  | 0.54 | 0.39-0.73 | **<0.001** |  | 0.53 | 0.38-0.73 | **<0.001** |  | 0.54 | 0.39-0.74 | **<0.001** |
| *Total population* |  |  |  |  |  |  |  |  |  |  |  |  |  |  |  |  |
| Strong ISS Men (Women=ref) |  | 1.21 | 0.80-1.83 | 0.365 |  | 1.23 | 0.82-1.86 | 0.323 |  | 1.32 | 0.87-2.01 | 0.178 |  | 1.33 | 0.87-2.03 | 0.187 |
| No frequent pain  (Frequent pain=ref) |  | 2.54 | 1.83-3.52 | **<0.001** |  | 2.44 | 1.76-3.38 | **<0.001** |  | 2.41 | 1.73-3.36 | **<0.001** |  | 2.24 | 1.60-3.13 | **<0.001** |
| Sex(male)*  Pain(no frequent) |  | 0.49 | 0.30-0.82 | **0.006** |  | 0.49 | 0.25-0.81 | **0.006** |  | 0.47 | 0.28-0.78 | **0.004** |  | 0.48 | 0.29-0.81 | **0.006** |
| Mixed ISS Men (Women=ref) |  | 1.18 | 0.76-1.81 | 0.459 |  | 1.19 | 0.77-1.84 | 0.462 |  | 1.19 | 0.77-1.84 | 0.438 |  | 1.19 | 0.77-1.84 | 0.439 |
| No frequent pain  (Frequent pain=ref) |  | 1.93 | 1.83-3.52 | **<0.001** |  | 1.87 | 1.33-2.64 | **<0.001** |  | 1.90 | 1.36-2.72 | **<0.001** |  | 1.82 | 1.28-2.57 | **0.001** |
| Sex(male)*  Pain(no frequent) |  | 0.45 | 0.30-0.82 | **0.003** |  | 0.45 | 0.26-0.77 | **0.003** |  | 0.45 | 0.26-0.76 | **0.003** |  | 0.46 | 0.27-0.78 | **0.005** |
| **Emotional social support** |  |  |  |  |  |  |  |  |  |  |  |  |  |  |  |  |
| *Frequent pain* |  |  |  |  |  |  |  |  |  |  |  |  |  |  |  |  |
| Strong ESS Men (Women=ref) |  | 0.51 | 0.34-0.75 | **0.001** |  | 0.51 | 0.34-0.76 | **0.001** |  | 0.50 | 0.33-0.75 | **0.001** |  | 0.43 | 0.31-0.61 | **<0.001** |
| Mixed ESS Men (Women=ref) |  | 1.04 | 0.64-1.71 | 0.865 |  | 1.05 | 0.64-1.72 | 0.849 |  | 0.99 | 0.60-1.64 | 0.978 |  | 0.81 | 0.53-1.24 | 0.329 |
| *No frequent pain* |  |  |  |  |  |  |  |  |  |  |  |  |  |  |  |  |
| Strong ESS Men (Women=ref) |  | 0.42 | 0.30-0.59 | **<0.001** |  | 0.43 | 0.31-0.60 | **<0.001** |  | 0.43 | 0.31-0.61 | **<0.001** |  | 0.50 | 0.33-0.75 | **0.001** |
| Mixed ESS Men (women=ref) |  | 0.80 | 0.53-1.21 | 0.295 |  | 0.81 | 0.53-1.22 | 0.308 |  | 0.81 | 0.53-1.24 | 0.329 |  | 1.00 | 0.60-1.65 | 0.989 |
| *Total population* |  |  |  |  |  |  |  |  |  |  |  |  |  |  |  |  |
| Strong ESS Men (Women=ref) |  | 0.51 | 0.34-0.75 | **0.001** |  | 0.51 | 0.34-0.77 | **0.001** |  | 0.52 | 0.35-0.78 | **0.001** |  | 0.51 | 0.34-0.77 | **0.001** |
| No frequent pain  (Frequent pain=ref) |  | 2.32 | 1.58-3.39 | **<0.001** |  | 2.23 | 1.53-3.27 | **<0.001** |  | 2.18 | 1.48-3.21 | **<0.001** |  | 2.09 | 1.42-3.08 | **<0.001** |
| Sex(male)*  Pain(no frequent) |  | 0.84 | 0.50-1.41 | 0.506 |  | 0.83 | 0.49-1.40 | 0.494 |  | 0.84 | 0.49-1.42 | 0.503 |  | 0.85 | 0.50-1.45 | 0.560 |
| Mixed ESS Men (women=ref) |  | 1.04 | 0.64-1.71 | 0.865 |  | 1.05 | 0.97-0.99 | 0.844 |  | 1.00 | 0.61-1.65 | 0.999 |  | 1.00 | 0.61-1.65 | 0.998 |
| No frequent pain  (Frequent pain=ref) |  | 1.32 | 0.82-2.12 | 0.260 |  | 1.29 | 0.64-1.73 | 0.294 |  | 1.25 | 0.77-2.02 | 0.369 |  | 1.25 | 0.77-2.03 | 0.370 |
| Sex(male)*  Pain(no frequent) |  | 0.77 | 0.40-1.47 | 0.422 |  | 0.77 | 0.80-2.08 | 0.419 |  | 0.82 | 0.43-1.57 | 0.548 |  | 0.82 | 0.43-1.57 | 0.546 |

Adjusted model 1: adjusted for age. Adjusted model 2: adjusted for age and level of education. Adjusted model 3: adjusted for age, level of education and place of birth ^1^OR=odds ratio, ^2^CI=confidence interval, ^3^ref=reference group
